# Causal relationship between the immune cells and ankylosing spondylitis: univariable, bidirectional, and multivariable Mendelian randomization

**DOI:** 10.3389/fimmu.2024.1345416

**Published:** 2024-04-09

**Authors:** Chaofan Qin, Qingshuai Yu, Zhongliang Deng, You Zhang, Mingxin Chen, Xin Wang, Tao Hu, Bo Lei, Zhengjian Yan, Si Cheng

**Affiliations:** Department of Orthopedics, Second Affiliated Hospital, Chongqing Medical University, Chongqing, China

**Keywords:** immune cells, ankylosing spondylitis, multivariable Mendelian randomization, univariable Mendelian randomization, bidirectional Mendelian randomization

## Abstract

**Background:**

Ankylosing spondylitis (AS) is an autoimmune disease that affects millions of individuals. Immune cells have been recognized as having a crucial role in the pathogenesis of AS. However, their relationship has not been fully explored.

**Methods:**

We chose to employ Mendelian randomization (MR) to investigate the potential correlation between immune cells and AS. We sourced the data on immune cells from the latest genome-wide association studies (GWASs). We obtained data on AS from the FinnGen consortium. Our comprehensive univariable MR analysis covered 731 immune cells to explore its potential causal relationship with AS. The primary analysis method was inverse-variance weighted (IVW). Additionally, we used Cochran’s Q test and the MR-Egger intercept test to assess the presence of pleiotropy and heterogeneity. We examined whether our results could be influenced by individual single-nucleotide polymorphisms (SNPs) using the leave-one-out test. We conducted a bidirectional MR to investigate the reverse relationship. We also applied multivariable MR to decrease the potential influence between the immune cells.

**Results:**

Overall, our univariable MR analysis revealed eight immune cells associated with AS. Among these, four immune cells contributed to an increased risk of AS, while four immune cells were identified as protective factors for AS. However, the Bonferroni test confirmed only one risk factor and one protective factor with a significance level of p < 6.84E−05. CD8 on effector memory CD8^+^ T cell could increase the risk of AS (p: 1.2302E−05, OR: 2.9871, 95%CI: 1.8289–4.8786). HLA DR on CD33^dim^ HLA DR^+^ CD11b^+^ could decrease the risk of AS (p: 1.2301E−06, OR: 0.5446, 95%CI: 0.4260–0.6962). We also identified a bidirectional relationship between CD4 on CD39^+^ activated CD4 regulatory T cells and AS utilizing the bidirectional MR. To address potential confounding among immune cells, we employed multivariable MR analysis, which revealed that only one immune cell had an independent effect on AS. HLA DR on CD33^dim^ HLA DR^+^ CD11b^+^ could decrease the risk of AS (p: 2.113E−06, OR: 0.0.5423, 95%CI: 0.4210–0.6983). Our findings were consistently stable and reliable.

**Conclusions:**

Our findings indicated a potential link between immune cells and AS, which could provide a new idea for future research. Nevertheless, the specific underlying mechanisms require further exploration.

## 1 Introduction

Ankylosing spondylitis (AS) is a chronic inflammatory disease with an incidence ranging from 1‰ to 3‰ in the general population (1). It is characterized by vertebral fusion, reduced mobility, and the potential for long-term disability in advanced stages (2, 3). Factors such as infection, environmental influences, and immune dysregulation have been suggested as potential triggers for AS (4–6). In recent years, the relationship between immunity and AS has garnered attention (3). Multiple immune cells are thought to be involved in the development of AS (7, 8). Regulatory T cells are lower in AS than in healthy individuals, leading to dysregulation of the immunity of the AS patient and thus to a decrease in the negative regulation of immunity in AS patients (9). However, the causal relationship between immunization and AS remains unclear and requires further investigation.

Mendelian randomization (MR) is a statistical method that uses genotype information as an instrumental variable (IV) to assess the causal relationship between exposure and outcome (10, 11). MR uses the Mendelian independent distribution law as a theoretical basis to explore the etiology of disease. Therefore, MR can effectively overcome the bias caused by confounding and reverse causation problems (10, 11). We plan to conduct an MR to investigate the relationship between immune cells and AS. This study mainly investigated the potential causal connection between immune cells and AS at a genetic level, utilizing univariable, bidirectional, and multivariable MR analyses.

## 2 Materials and methods

### 2.1 Study design

**Figure 1:**
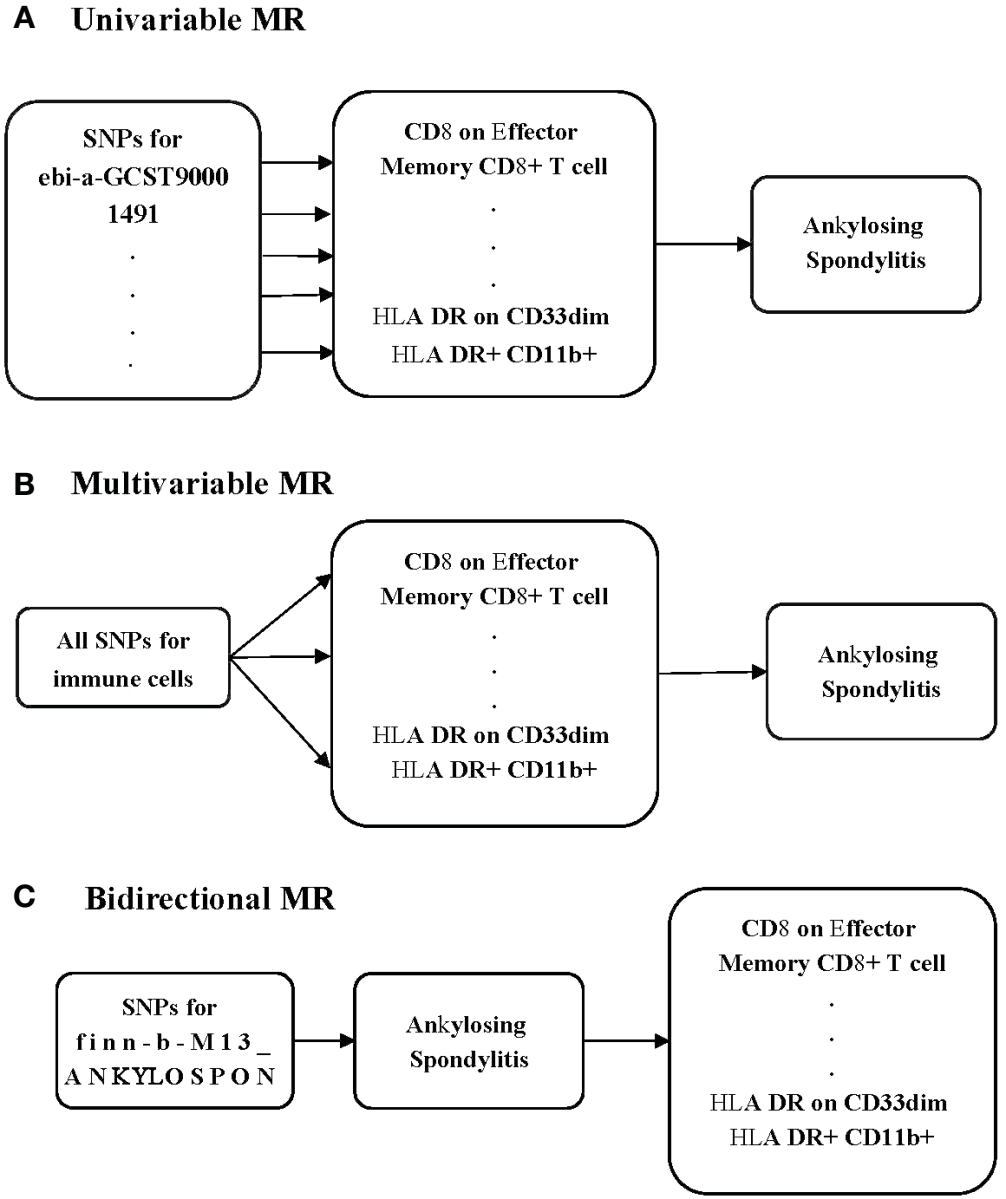
Schematic presentation of **(A)** univariable, **(B)** multivariable, and **(C)** bidirectional.

To investigate the potential causal relationship between immune cells and AS, we conducted a univariable MR analysis in our study (**Figure 1A**). All genetic variations used as IVs adhere to the three fundamental assumptions: 1) the chosen IVs had a clear association with the exposure, 2) the selected IVs had no relationship with any confounding factors, and 3) the IVs could influence the outcomes solely through their impact on the exposure (12). To minimize the potential for reverse connection, we conducted a bidirectional MR analysis to explore the relationship between immune cells and AS (**Figure 1B**). Recognizing that various immune cells may interact and affect each other due to genetic pleiotropy, potentially introducing confounding effects, we subsequently carried out a multivariable MR analysis to assess the direct influence of immune cells on AS (**Figure 1C**).

### 2.2 GWAS data source

The genome-wide association study (GWAS) data on immune cells were acquired from the latest study involving 3,757 individuals of Sardinian descent within the European population (13). This study included 3,757 cases and 3,027 controls, with a gender distribution of 43% male and 57% female. The ages of the participants ranged from 18 to 102 years (13). A total of 731 immunophenotypes were included, including 118 absolute cell (AC) counts, 389 median fluorescence intensities (MFIs) reflecting surface antigen levels, 32 morphological parameters (MPs), and 192 relative cell (RC) counts (13). The MFI, AC, and RC features encompass B cells, CDCs, mature stages of T cells, monocytes, myeloid cells, TBNK (T cells, B cells, and natural killer cells), and Treg panels, while the MP feature encompasses CDC and TBNK panels (13). This study measured 22 million genetic variations (13).

The data related to AS were sourced from the FinnGen database (https://www.finngen.fi/fi). This dataset encompasses 1,462 cases, 164,682 controls, and 16,380,022 single-nucleotide polymorphisms (SNPs). Diagnoses of AS were made in accordance with the International Classification of Diseases, specifically ICD-10 (M45), ICD-9 (7200), and ICD-8 (7124) coding standards. All populations included in the database were of European origin. Additional relevant information can be obtained from the FinnGen website (Risteys · Home (finregistry.fi)).

### 2.3 Selection of instrumental variables

We conducted various strict quality controls to choose IVs to satisfy the three core assumptions of MR analysis and ensure the robustness and reliability of MR analysis. First, we selected the SNPs of immune cells at a genome-wide significance threshold (p < 5E−08) (10). Second, we addressed the issue of linkage disequilibrium (LD) between SNPs by removing strongly linked variants (r^2^ = 0.001, clumping distance of 10,000 kb). This step aimed to mitigate any potential bias in the results caused by LD (10). Then, we utilized the PhenoScanner database to exclude the effect of confounder factors. Finally, we computed the F-statistics for all the selected SNPs. We excluded SNPs with F-statistics less than 10 to ensure that all remaining SNPs were strongly associated with the exposure (14). We calculated F-statistics using the formula F = beta^2^/standard error^2^(SE) (15, 16) (**Supplementary Table 1**).

### 2.4 Statistical analysis

In our MR analysis, we employed the inverse-variance weighted (IVW) method as our primary analytical approach. We established statistical significance by setting the threshold for p-values at 6.84E−05, which was adjusted using the Bonferroni method (0.05/731). Additionally, we considered p-values falling between 6.84E−05 and 0.05 suggestive of significance. Since the IVW method assumes the absence of an intercept term, we conducted the MR-Egger test to assess the presence of the intercept (17). Additionally, to enhance the robustness of our results, we employed the MR-Egger, weighted median, weighted mode, and simple mode methods.

pWe performed Cochran’s Q test and MR-Egger intercept analysis to ensure the absence of heterogeneity and pleiotropy. Heterogeneity was deemed present if the Q–p-value was less than 0.05, leading to the utilization of a random effects model for the analysis (18). MR-Egger intercept p-value exceeding 0.05 indicated the absence of pleiotropy (19). Finally, we employed leave-one-out analysis to investigate the potential impact of single SNP points on the causal relationship between immune cells and AS.

We conducted all statistical analyses using the “TwoSampleMR” (version 0.5.7) packages within the R statistical software (version 4.3.1).

## 3 Result

### 3.1 Univariable MR

In our study, we investigated the association between 731 immune cells and AS (**Supplementary Table 2**). Our analysis revealed that 39 of these immune cells were significantly associated with AS, comprising 22 risk factors and 17 protective factors (**Supplementary Table 3**). However, we excluded 31 factors with fewer than three SNPs each due to limitations in the number of available SNPs. Consequently, we identified four potential pathogenic factors and four potential protective factors. We calculated terminally differentiated CD8^+^ T cell %CD8^+^ T cell, HLA DR^+^ CD8^+^ T cell absolute count, and HLA DR^+^ CD8^+^ T cell %lymphocyte using the fixed effects model (**Table 1**). For HLA DR on CD14^−^ CD16^+^ monocyte, CD8 on effector memory CD8^+^ T cell, CD4 on CD39^+^ activated CD4 regulatory T cell, HLA DR on CD33^dim^ HLA DR^+^ CD11b^+^, and HLA DR on CD33^dim^ HLA DR^+^ CD11b^−^, we employed a random effects model for calculation (**Table 1**).

**Table 1:** The result of the univariable MR.

Exposure	Method	nSNP	pval	OR	95%CI
Terminally differentiated CD8^+^ T cell %CD8^+^ T cell	IVW	3	0.0367	1.2703	1.0149–1.59
HLA DR^+^ CD8^+^ T cell absolute count	IVW	4	0.0143	1.2263	1.0416–1.4438
HLA DR^+^ CD8^+^ T cell %lymphocyte	IVW	3	0.0414	1.2355	1.0082–1.5139
HLA DR on CD14^−^ CD16^+^ monocyte	IVW: random effects	7	0.0016	0.7507	0.6280–0.8973
CD8 on effector memory CD8^+^ T cell	IVW: random effects	4	1.23E−05	2.9871	1.8289–4.8786
CD4 on CD39^+^ activated CD4 regulatory T cell	IVW: random effects	4	0.0386	0.1793	0.0352–0.9141
HLA DR on CD33^dim^ HLA DR^+^ CD11b^+^	IVW: random effects	4	1.23E−06	0.5446	0.426–0.6962
HLA DR on CD33^dim^ HLA DR^+^ CD11b^−^	IVW: random effects	5	0.0054	0.4689	0.2749–0.7997

IVW, inverse-variance weighted; OR, odds ratio; CI, confidence interval; MR, Mendelian randomization.

**Figure 2:**
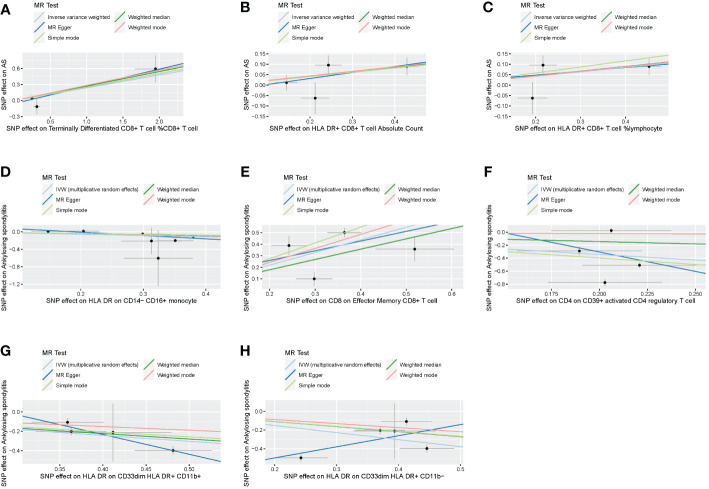
Scatter plots of effects of immune cells on the AS using univariable MR. **(A)** Terminally differentiated CD8^+^ T cell %CD8^+^ T cell. **(B)** HLA DR^+^ CD8^+^ T cell absolute count. **(C)** HLA DR^+^ CD8^+^ T cell %lymphocyte. **(D)** HLA DR on CD14^−^ CD16^+^ monocyte. **(E)** CD8 on effector memory CD8^+^ T cell. **(F)** CD4 on CD39^+^ activated CD4 regulatory T cell. **(G)** HLA DR on CD33^dim^ HLA DR^+^ CD11b^+^. **(H)** HLA DR on CD33^dim^ HLA DR^+^ CD11b^−^. AS, ankylosing spondylitis; MR, Mendelian randomization.

**Figure 3:**
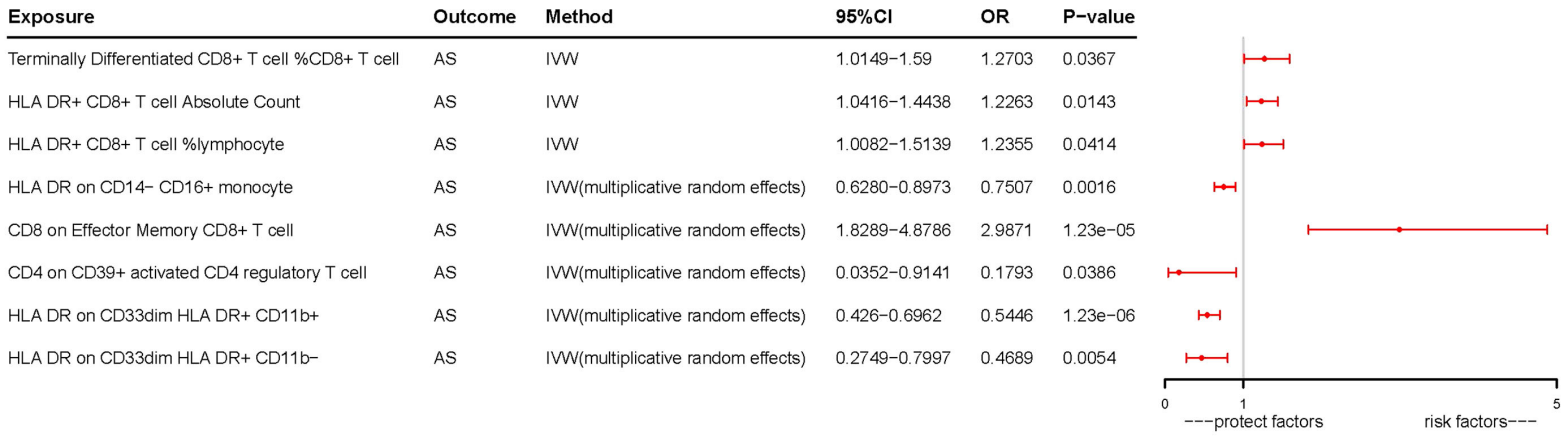
Forest plots of effects of immune cells on the AS using univariable MR. AS, ankylosing spondylitis; IVW, inverse-variance weighted; OR, odds ratio; CI, confidence interval; MR, Mendelian randomization.

Following the application of the Bonferroni test, we identified one risk factor and one protective factor associated with AS (**Table 1**; **Figures 2**, **3**). CD8 on effector memory CD8^+^ T cell could significantly increase the risk of AS (p: 1.2302E−05, OR: 2.9871, 95%CI: 1.8289–4.8786). HLA DR on CD33^dim^ HLA DR^+^ CD11b^+^ could significantly decrease the risk of AS (p: 1.2301E−06, OR: 0.5446, 95%CI: 0.4260–0.6962).

### 3.2 Bidirectional MR

**Figure 4:**
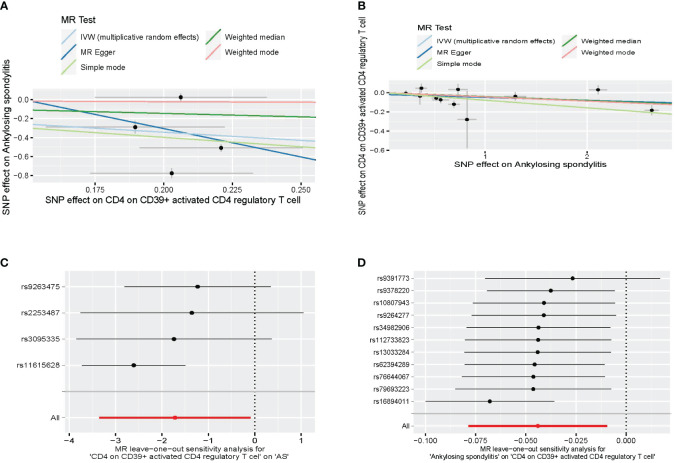
The result of bidirectional MR between CD4 on CD39^+^ activated CD4 regulatory T cell and AS. **(A)** The scatter plot of CD4 on CD39^+^ activated CD4 regulatory T cell on AS. **(B)** The scatter plot of AS on CD4 on CD39^+^ activated CD4 regulatory T cell. **(C)** The leave-one-out plot of CD4 on CD39^+^ activated CD4 regulatory T cell on AS. **(D)** The leave-one-out plot of AS on CD4 on CD39^+^ activated CD4 regulatory T cell. MR, Mendelian randomization; AS, ankylosing spondylitis.

**Figure 5:**
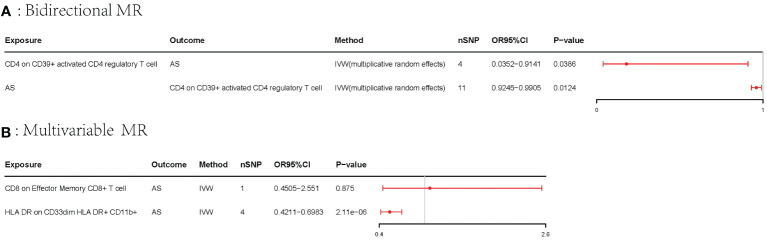
Forest plots of the bidirectional MR and multivariable MR. AS, ankylosing spondylitis; IVW, inverse-variance weighted; OR, odds ratio; CI, confidence interval; MR, Mendelian randomization. **(A)** Forest plots of bidirectional MR. **(B)** Forest plots of the multivariable MR.

A bidirectional MR analysis was performed, and evidence of a reverse relationship was identified, particularly between CD4 on CD39^+^ activated CD4 regulatory T cells and AS. The bidirectional MR was analyzed by the random effects model. It was observed that AS could lead to a decrease in CD4 on CD39^+^ activated CD4 regulatory T cell (p: 0.0124, OR: 0.9570, 95%CI: 0.9245–0.9905) (**Table 2**; **Figures 4**, **5A**). The potential causal relationship between them appears to be bidirectional.

**Table 2:** The result of the bidirectional MR.

Exposure	Method	nSNP	pval	OR	95%CI
CD4 on CD39^+^ activated CD4 regulatory T cell	IVW: random effects	4	0.0386	0.1793	0.0352–0.9141
AS	IVW: random effects	11	0.0124	0.9570	0.9245–0.9905

AS, ankylosing spondylitis; IVW, inverse-variance weighted; OR, odds ratio; CI, confidence interval; MR, Mendelian randomization.

### 3.3 Multivariable MR

The multivariable MR analysis included HLA DR on CD33^dim^ HLA DR^+^ CD11b^+^ and CD8 on effector memory CD8^+^ T cell. HLA DR on CD33^dim^ HLA DR^+^ CD11b^+^ could decrease the risk of AS (p: 2.113E−06, OR: 0.0.5423, 95%CI: 0.4210–0.6983) (**Table 3**; **Figure 5B**).

**Table 3:** The result of the multivariable MR.

Exposure	Method	nSNP	pval	OR	95%CI
CD8 on effector memory CD8^+^ T cell	IVW	1	0.8750	1.0720	0.4505–2.551
HLA DR on CD33^dim^ HLA DR^+^ CD11b^+^	IVW	4	2.11E−06	0.5422	0.4211–0.6983

IVW, inverse-variance weighted; OR, odds ratio; CI, confidence interval; MR, Mendelian randomization.

### 3.4 Sensitivity analyses

#### 3.4.1 Univariable MR

**Figure 6:**
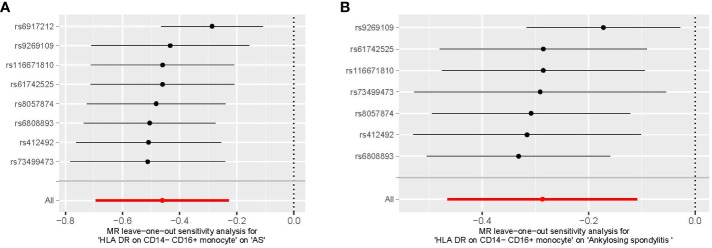
The leave-one-out plot of HLA DR on CD14^−^ CD16^+^ monocyte on AS. **(A)** The leave-one-out plot of HLA DR on CD14^−^ CD16^+^ monocyte on AS with the rs6917212. **(B)** The leave-one-out plot of HLA DR on CD14^−^ CD16^+^ monocyte on AS without the rs6917212. AS, ankylosing spondylitis.

**Figure 7:**
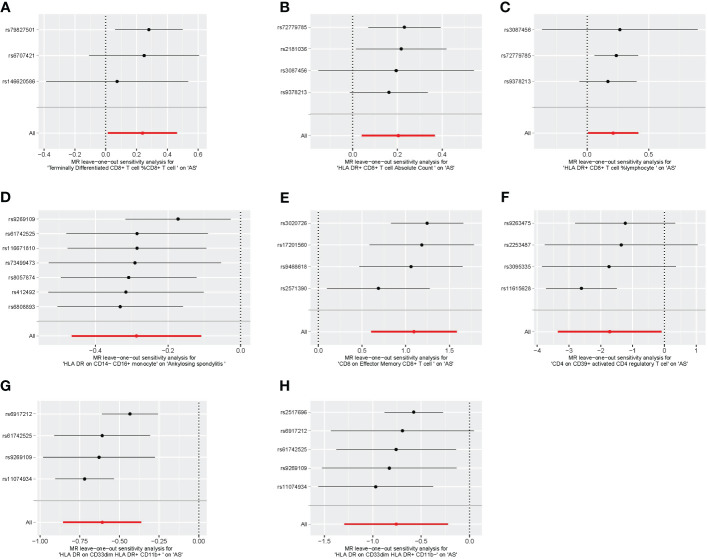
The leave-one-out plot of univariable MR. **(A)** Terminally differentiated CD8^+^ T cell %CD8^+^ T cell. **(B)** HLA DR^+^ CD8^+^ T cell absolute count. **(C)** HLA DR^+^ CD8^+^ T cell %lymphocyte. **(D)** HLA DR on CD14^−^ CD16^+^ monocyte. **(E)** CD8 on effector memory CD8^+^ T cell. **(F)** CD4 on CD39^+^ activated CD4 regulatory T cell. **(G)** HLA DR on CD33^dim^ HLA DR^+^ CD11b^+^. **(H)** HLA DR on CD33^dim^ HLA DR^+^ CD11b^−^. MR, Mendelian randomization.

We conducted Cochran’s Q test and MR-Egger intercept test to assess the robustness of our results. Notably, we observed that only the MR-Egger intercept test p-value for HLA DR on CD14^−^ CD16^+^ monocyte was less than 0.05, indicating the presence of horizontal pleiotropy (**Table 4**). During the leave-one-out test, we identified an anomaly with the SNP (rs6917212) (**Figure 6**). As a result, we chose to remove this SNP to ensure the stability of our results. Through this adjustment, horizontal pleiotropy for this exposure was no longer present, reinforcing the validity of our decision (**Table 4**). We found no evidence of horizontal pleiotropy in other analyses of immune cells (**Table 5**). Heterogeneity was not found among terminally differentiated CD8^+^ T cell %CD8^+^ T cell, HLA DR^+^ CD8^+^ T cell absolute count, and HLA DR^+^ CD8^+^ T cell %lymphocyte (**Table 5**). However, the p-values of Cochran’s Q test were less than 0.05 for HLA DR on CD14^−^ CD16^+^ monocyte, CD8 on effector memory CD8^+^ T, CD4 on CD39^+^ activated CD4 regulatory T cell, HLA DR on CD33^dim^ HLA DR^+^ CD11b^+^, and HLA DR on CD33^dim^ HLA DR^+^ CD11b^−^. These results indicated the presence of heterogeneity (**Table 5**). Therefore, we employed the random effects model to analyze the effects on AS (**Table 1**, **Figure 2**). Furthermore, the leave-one-out test revealed that the association between immune cells and AS remained unaffected by the exclusion of individual SNP (**Figure 7**). In summary, our results have been validated and are considered reliable and acceptable.

**Table 4:** The result of MR-Egger intercept test of HLA DR on CD14^−^ CD16^+^ monocyte with or without the SNP of rs6917212.

Exposure	egger_intercept	pval
HLA DR on CD14^−^ CD16^+^ monocyte with rs6917212	0.2273	0.0227
HLA DR on CD14^−^ CD16^+^ monocyte without rs6917212	0.1404	0.0853

MR, Mendelian randomization; SNP, single-nucleotide polymorphism.

**Table 5:** The sensitivity analyses of the univariable MR.

Exposure	egger_intercept	pval	Q	Q_pval
Terminally differentiated CD8^+^ T cell %CD8^+^ T cell	−0.0568	0.6421	2.2412	3.26E−01
HLA DR^+^ CD8^+^ T cell absolute count	−0.0257	0.7409	3.2385	3.56E−01
HLA DR^+^ CD8^+^ T cell %lymphocyte	0.012	0.9469	3.1202	2.10E−01
HLA DR on CD14^−^ CD16^+^ monocyte	0.1404	0.0853	37.3293	4.06E−06
CD8 on effector memory CD8^+^ T cell	0.0909	0.885	25.6061	1.15E−05
CD4 on CD39^+^ activated CD4 regulatory T cell	0.8896	0.8542	159.9431	1.89E−34
HLA DR on CD33^dim^ HLA DR^+^ CD11b^+^	0.5791	0.0857	12.9616	4.72E−03
HLA DR on CD33^dim^ HLA DR^+^ CD11b^−^	−0.7377	0.1163	96.1627	6.45E−20

MR, Mendelian randomization.

#### 3.4.2 Bidirectional MR

We found heterogeneity in the bidirectional MR (**Table 6**). Therefore, we decided to use the random effects model (**Table 2**, **Figure 4**). The horizontal pleiotropy was absent in the bidirectional MR (**Table 6**). Meanwhile, the leave-one-out test indicated that the results were not decided by a single SNP (**Figure 4**).

**Table 6:** The sensitivity analyses of the bidirectional MR.

Exposure	egger_intercept	pval	Q	Q_pval
CD4 on CD39^+^ activated CD4 regulatory T cell	0.8896	0.8542	159.9431	1.89E−34
Ankylosing spondylitis	−0.0191	0.5326	21.4395	0.0182

MR, Mendelian randomization.

## 4 Discussion

In this study, we employed univariable, bidirectional, and multivariable MR to explore the association between 731 immune cells and AS. Using the extensive public GWAS summary data, we uncovered the complex relationship between immune cells and AS. Our findings were confirmed by sensitivity analysis. Additionally, we used the Bonferroni test to further support the association between immune cells and AS. Through univariable MR coupled with the Bonferroni test, we validated one risk factor and one protective factor. Finally, we have conclusively demonstrated that HLA-DR expression on CD33^dim^ HLA-DR^+^ CD11b^+^ cells decreases the risk of ankylosing spondylitis through multivariable MR, after controlling for the effect between immune cells. Employing bidirectional MR, we revealed a bidirectional link between immune cells and AS.

### 4.1 Univariable MR

Our findings indicated that HLA-DR exhibits both protective and promotive effects on AS across various immune cells. It is hypothesized that the presence of different HLA-DR subtypes on various immune cells determines their respective roles. Kchir reported elevated expression levels of HLA-DRB1*11 in AS patients, and HLA-DRB1*11 did not have a direct effect on AS but showed dependence on HLA-B27 (20). Meanwhile, their findings also indicated that HLA-DRB1*13 played a protective role in AS (20). These findings support our results on the role of HLA-DR in AS.

Our study demonstrated that CD8^+^ T cells can contribute to the development of AS. Terminally differentiated CD8^+^ T cell %CD8^+^ T cell, HLA DR^+^ CD8^+^ T cell absolute count, HLA DR^+^ CD8^+^ T cell %lymphocyte, and CD8 on effector memory CD8^+^ T cell could increase the risk of AS. Previous studies have shown a significant increase in IL-6 levels in CD8^+^ T cells in the peripheral blood of patients with AS (9). IL-6, a pro-inflammatory cytokine, may enhance the body’s inflammatory response and promote the progression of AS (9, 21). The role of CD8^+^ cells in the pathogenesis of AS is dependent on the presentation of antigens through HLA-B27, and it is widely acknowledged that HLA-B27 plays a significant role in promoting the development of AS (22–25). Moreover, cytotoxic cells restricted by HLA-B27 can be found in the synovial fluids of AS patients (26).

In this study, we also found that HLA DR on CD14^−^ CD16^+^ monocytes was associated with a reduced risk of AS. Wright proposed that CD14^−^ CD16^+^ mononuclear cells may contribute to AS by promoting T helper 17 cells (Th17) responses, which can produce the IL-17 to accelerate the pathogenesis of AS (24, 27–29). However, our findings differed from those reported by Wright. It is proposed that the protective influence exerted by HLA-DR offsets the promotional impact of CD14^−^ CD16^+^ on AS. The protective influence of HLA-DR has been reported by a previous study (20).

### 4.2 Bidirectional MR

CD4 on CD39^+^ activated CD4 regulatory T cell has the capacity to decrease the risk of AS. Conversely, AS can diminish the population of CD4 on CD39^+^ activated CD4 regulatory T cells. However, CD4 cell activation leads to increased secretion of IL-10, which exerts an anti-inflammatory effect and diminishes the risk of AS (9, 21). However, the body experiences immune dysregulation in AS patients, impairing immune suppression function and potentially resulting in reduced IL-10 secretion by CD4 cells, thereby inhibiting their function (9). The collaborative action of CD39 and CD73 results in the conversion of ATP to ADP and cAMP, ultimately generating adenosine (30, 31). Adenosine can interact with multiple receptors, including A1, A2A, A2B, and A3, leading to various immune responses (31, 32). Adenosine stimulates immune responses through A1 and A3 receptors, while it exerts immunosuppressive effects when engaging with A2A and A2B receptors (32–34). In the context of AS pathogenesis, we hypothesize that CD39 utilizes the latter pathway to participate in AS.

### 4.3 Multivariable MR

First, we identified potential causative factors of AS through univariable MR. The use of the Bonferroni test reduced the probability of a type I error and increased the stability of our results. Furthermore, to eliminate mutual confounding between immune cells, we employed multivariate MR. These methods have significantly reduced the influence of confounding factors and greatly increased result confidence. It has been confirmed that the development of AS can be inhibited by HLA-DR on CD33^dim^ HLA DR^+^ CD11b^+^.

It is hypothesized that HLA-DR reduces the risk of AS through its subtype HLA-DRB113. Previous studies have indicated decreased expression of HLA-DRB113 in AS patients (20). Further investigations are necessary to determine if other isoforms may also contribute. Furthermore, AS patients with low activity were found to have decreased CD11b expression compared to normal controls (35). This may be due to CD11b’s ability to inhibit the inflammatory response, thereby reducing the occurrence of AS. In mice, CD11b promotes neutrophil apoptosis to inhibit inflammation (36). It is suspected that a similar effect of reducing inflammation may occur in the human body. Additionally, CD33 inhibits the expression of pro-inflammatory cytokines, including IL-1β and TNF-α (37, 38). Consequently, CD33 may decrease the incidence of AS by mitigating the body’s inflammatory response. However, further research is required to elucidate the specific mechanisms.

Our MR analysis has several strengths. First, we used a comprehensive approach, including univariable, bidirectional, and multivariable MR, to address potential confounding factors and reverse causality. Second, we conducted multiple sensitivity analyses to validate our hypotheses and minimize bias. Third, our research was limited to the European population to reduce population bias. Finally, we used the Bonferroni test to confirm the causative role of AS.

However, it is important to note that our study has limitations. The conclusions drawn from our data could not be immediately generalized to other populations, as all of our data originated from European sources. Additionally, the relatively small sample size may introduce bias, highlighting the need for larger samples to ensure more robust results.

## 5 Conclusion

Our extensive MR analysis has unveiled the intricate relationship between immune cells and AS. These immune cells can serve as both contributing and protective factors, opening new perspectives for the treatment and prevention of AS. However, the specific mechanisms behind this phenomenon have yet to be fully explored. Therefore, further experiments are necessary to elucidate these underlying mechanisms, and our study can serve as a guiding foundation for future research.

## Data availability statement

Publicly available datasets were analyzed in this study. This data can be found here: https://gwas.mrcieu.ac.uk/.

## Author contributions

CQ: Data curation, Methodology, Validation, Visualization, Writing – original draft, Writing – review & editing, Software. QY: Conceptualization, Methodology, Writing – review & editing, Supervision. ZD: Writing – review & editing, Supervision, Project administration, Methodology. YZ: Conceptualization, Methodology, Writing – review & editing, Writing – original draft, Data curation. MC: Conceptualization, Methodology, Supervision, Writing – review & editing. XW: Writing – review & editing. TH: Writing – review & editing. BL: Writing – review & editing. ZY: Conceptualization, Supervision, Writing – review & editing. SC: Writing – review & editing, Validation, Supervision, Conceptualization.
