# Supplementary material for: Causal relationship between the immune cells and ankylosing spondylitis: univariable, bidirectional, and multivariable Mendelian randomization
Source: Front Immunol. 2024 Apr 9;15:1345416. doi: 10.3389/fimmu.2024.1345416 (PMC11035830; doi:10.3389/fimmu.2024.1345416)
Supplement: Supplementary file 2 [file DataSheet_2.pdf]

STROBE-MR checklist of recommended items to address in reports of Mendelian randomization studies1 2

Item No.	Section		Checklist item	Page No.	Relevant text from manuscript
1	**TITLE and ABSTRACT**		Indicate Mendelian randomization (MR) as the study's design in the title and/or the abstract if that is a main purpose of the study	1	Causal Relationship Between the Immune Cells and Ankylosing Spondylitis: Univariable, Bidirectional and Multivariable Mendelian Randomization
	**INTRODUCTION**				
2	**Background**		Explain the scientific background and rationale for the reported study. What is the exposure? Is a potential causal relationship between exposure and outcome plausible? Justify why MR is a helpful method to address the study question	2	Ankylosing spondylitis (AS) is an autoimmune disease that affects millions of individuals. Immune cells have been recognized as a crucial role in the pathogenesis of AS. However, the relationship has not been fully explored.
3	**Objectives**		State specific objectives clearly, including pre-specified causal hypotheses (if any). State that MR is a method that, under specific assumptions, intends to estimate causal effects	2	We conducted a MR study to investigate the possible aetiology of AS. In this study, immune cells were used as the exposure and AS as the outcome. We comprehensively analysed the relationship between 731 immune cells and AS.
	**METHODS**				
4	**Study design and data sources**		Present key elements of the study design early in the article. Consider including a table listing sources of data for all phases of the study. For each data source contributing to the analysis, describe the following:		
		a)	Setting: Describe the study design and the underlying population, if possible. Describe the setting, locations, and relevant dates, including periods of recruitment, exposure, follow-up, and data collection, when available.	2<br>3	Setting: see Figure 1 for a schematic summary of the study design.<br>Exposure: 731 immune cells, supplementary Table2.<br>Outcome: the data for AS was sourced from the FinnGen database. Cases and controls were defined based on ICD-10 (M45), ICD-9 (7200), and ICD-8 (7124) coding standards.
		b)	Participants: Give the eligibility criteria, and the sources and methods of selection of participants. Report the sample size, and whether any power or sample size calculations were carried out prior to the main analysis		
		c)	Describe measurement, quality control and selection of genetic variants	3	To exclude bias from weak instrumental variables, we used strict inclusion criteria for SNPs.<br>Firstly, the SNPs of immune cells were selected at a genome-wide significance threshold (P<5E-08).

					Secondly, we addressed the issue of linkage disequilibrium (LD) between SNPs by removing strongly linked variants ($r^2 = 0.001$, clumping distance of 10,000 kb). SNPs with F-statistics less than 10 were excluded
		d)	For each exposure, outcome, and other relevant variables, describe methods of assessment and diagnostic criteria for diseases		Exposure: 731 immune cells, supplementary Table2.<br>Outcome: the data for AS was sourced from the FinnGen database. Cases and controls were defined based on ICD-10 (M45), ICD-9 (7200), and ICD-8 (7124) coding standards.
		e)	Provide details of ethics committee approval and participant informed consent, if relevant		All participants provided informed consent in all the corresponding original studies. All data used in this work are publicly available from studies with relevant participant consent and ethical approval. Ethical approval from an institutional review board was not necessary for the present study as only publicly available summary level data was used.
5	**Assumptions**		Explicitly state the three core IV assumptions for the main analysis (relevance, independence and exclusion restriction) as well assumptions for any additional or sensitivity analysis	2<br>3	Three key assumptions explained<br>Sensitivity analyses explained
6	**Statistical methods: main analysis**		Describe statistical methods and statistics used		
		a)	Describe how quantitative variables were handled in the analyses (i.e., scale, units, model)		NA
		b)	Describe how genetic variants were handled in the analyses and, if applicable, how their weights were selected		NA
		c)	Describe the MR estimator (e.g. two-stage least squares, Wald ratio) and related statistics. Detail the included covariates and, in case of two-sample MR, whether the same covariate set was used for adjustment in the two samples		IVW<br>Both exposure and outcome GWASs were adjusted for sex, age, and PCs.
		d)	Explain how missing data were addressed		NA
		e)	If applicable, indicate how multiple testing was addressed	3	P values < 0.05 were considered nominally significant, whereas high-confidence findings were those that survived a stringent Bonferroni correction to account for multiple testing (731 immune cells), setting the following threshold for statistical significance: P=0.05/731=6.84E-05.

7	**Assessment of assumptions**	Describe any methods or prior knowledge used to assess the assumptions or justify their validity	3	Only independent SNPs ($r2 < 0.001$ within 10,000 kb windows), strongly associated ($P \leq 5E-08$) with the immune cells, were used.
8	**Sensitivity analyses and additional analyses**	Describe any sensitivity analyses or additional analyses performed (e.g. comparison of effect estimates from different approaches, independent replication, bias analytic techniques, validation of instruments, simulations)	3	For exposures that were significantly associated with an outcome, we looked up each exposure SNP in the Phenoscanner to evaluate whether the association could be due to pleiotropy.
			3	We performed the MR-Egger intercept analysis to ensure the absence pleiotropy. Besides IVW, we also employed the MR-Egger, weighted median, weighted mode, and simple mode methods.
			3	We conducted the Cochran's Q statistical test to assess heterogeneity
			3	We performed leave-one-out analyses to examine the robustness of the IVW estimates and whether any specific SNP drove the association.
			2	Recognizing that various immune cells might interact and affect each other due to genetic pleiotropy, potentially introducing confounding effects, we subsequently carried out a multivariable MR analysis to assess the direct influence of immune cells on AS.
9	**Software and pre-registration**			
		a) Name statistical software and package(s), including version and settings used	3	We conducted all statistical analyses using the "TwoSampleMR" (version 0.5.7) packages within the R statistical software (version 4.3.1).
		b) State whether the study protocol and details were pre-registered (as well as when and where)		No.
	**RESULTS**			
10	**Descriptive data**			
		a) Report the numbers of individuals at each stage of included studies and reasons for exclusion. Consider use of a flow diagram		Fig1.

		b)	Report summary statistics for phenotypic exposure(s), outcome(s), and other relevant variables (e.g. means, SDs, proportions)	2-3	Exposures: 731 immune cells (Supplementary Table2).<br>Outcomes: ankylosing spondylitis. Additional relevant information can be obtained from the FinnGen website (Risteys · Home (finregistry.fi)).
		c)	If the data sources include meta-analyses of previous studies, provide the assessments of heterogeneity across these studies		No.
		d)	For two-sample MR:<br>i. Provide justification of the similarity of the genetic variant-exposure associations between the exposure and outcome samples<br>ii. Provide information on the number of individuals who overlap between the exposure and outcome studies	4-5	We calculated the Cochran's Q-test for heterogeneity to assess heterogeneity across the cohorts and found minimal heterogeneity for the included variants for the outcomes.<br>Due to the use of summary-level statistics we were not able to identify individuals who overlap between the immune cells and AS.
11	**Main results**				
		a)	Report the associations between genetic variant and exposure, and between genetic variant and outcome, preferably on an interpretable scale	3	The SNPs of immune cells were selected at a genome-wide significance threshold (P<5E-08). we addressed the issue of linkage disequilibrium (LD) between SNPs by removing strongly linked variants ($r^2 = 0.001$, clumping distance of 10,000 kb).
		b)	Report MR estimates of the relationship between exposure and outcome, and the measures of uncertainty from the MR analysis, on an interpretable scale, such as odds ratio or relative risk per SD difference	3	The SNPs of immune cells were selected at a genome-wide significance threshold (P<5E-08). we addressed the issue of linkage disequilibrium (LD) between SNPs by removing strongly linked variants ($r^2 = 0.001$, clumping distance of 10,000 kb).
		c)	If relevant, consider translating estimates of relative risk into absolute risk for a meaningful time period		NA.
		d)	Consider plots to visualize results (e.g. forest plot, scatterplot of associations between genetic variants and outcome versus between genetic variants and exposure)		Fig3, Fig5.
12	**Assessment of assumptions**				
		a)	Report the assessment of the validity of the assumptions		Methods to assess the robustness of MR findings: MR Egger, weighted median, simple mode, weighted mode, Cochran's Q statistical test, PhenoScanner, leave-one-out analyses and post-hoc analyses.

		b)	Report any additional statistics (e.g., assessments of heterogeneity across genetic variants, such as I^2, Q statistic or E-value)	4-5	For the MR analyses, heterogeneity was reported for all analyses consisting of ≥ 2 SNPs (table 5 and table 6).
13	**Sensitivity analyses and additional analyses**				
		a)	Report any sensitivity analyses to assess the robustness of the main results to violations of the assumptions	3	The SNPs of immune cells were selected at a genome-wide significance threshold (P<5E-08). we addressed the issue of linkage disequilibrium (LD) between SNPs by removing strongly linked variants (r^2 = 0.001, clumping distance of 10,000 kb).
		b)	Report results from other sensitivity analyses or additional analyses		NA.
		c)	Report any assessment of direction of causal relationship (e.g., bidirectional MR)	4	We conducted a bi-directional two-sample MR analysis using AS as the exposure and immune cells as the outcome. We found that AS affected CD4 on $CD39^+$ activated CD4 regulatory T cells (table2).
		d)	When relevant, report and compare with estimates from non-MR analyses		NA
		e)	Consider additional plots to visualize results (e.g., leave-one-out analyses)		Fig4 and fig7.
	**DISCUSSION**				
14	**Key results**		Summarize key results with reference to study objectives	5,6	Our extensive MR analysis has unveiled the intricate relationship between immune cells and AS. These immune cells can serve as both contributing and protective factors.
15	**Limitations**		Discuss limitations of the study, taking into account the validity of the IV assumptions, other sources of potential bias, and imprecision. Discuss both direction and magnitude of any potential bias and any efforts to address them	7	The conclusions drawn from our data could not be immediately generalized to other populations, as all of our data originates from European sources. Additionally, the relatively small sample size may introduce bias, highlighting the need for larger samples to ensure more robust results.
16	**Interpretation**				
		a)	Meaning: Give a cautious overall interpretation of results in the context of their limitations and in comparison with other studies	5-7	
		b)	Mechanism: Discuss underlying biological mechanisms that could drive a potential causal relationship between the investigated exposure and the outcome, and whether	5-7	

			the gene-environment equivalence assumption is reasonable. Use causal language carefully, clarifying that IV estimates may provide causal effects only under certain assumptions		
		c)	Clinical relevance: Discuss whether the results have clinical or public policy relevance, and to what extent they inform effect sizes of possible interventions	5-7	
17	**Generalizability**		Discuss the generalizability of the study results (a) to other populations, (b) across other exposure periods/timings, and (c) across other levels of exposure	6-7	The conclusions drawn from our data could not be immediately generalized to other populations, as all of our data originates from European sources.
	**OTHER INFORMATION**				
18	**Funding**		Describe sources of funding and the role of funders in the present study and, if applicable, sources of funding for the databases and original study or studies on which the present study is based		Our study was supported by Chongqing Medical Scientific Research Project (Grant No. 2024WSJK057).
19	**Data and data sharing**		Provide the data used to perform all analyses or report where and how the data can be accessed, and reference these sources in the article.	2-3	Sources of data and basic information are presented in the manuscript.
20	**Conflicts of Interest**		All authors should declare all potential conflicts of interest		The authors declare that they have no competing interests

This checklist is copyrighted by the Equator Network under the Creative Commons Attribution 3.0 Unported (CC BY 3.0) license.

1. Skrivankova VW, Richmond RC, Woolf BAR, Yarmolinsky J, Davies NM, Swanson SA, et al. Strengthening the Reporting of Observational Studies in Epidemiology using Mendelian Randomization (STROBE-MR) Statement. JAMA. 2021;under review.

2. Skrivankova VW, Richmond RC, Woolf BAR, Davies NM, Swanson SA, VanderWeele TJ, et al. Strengthening the Reporting of Observational Studies in Epidemiology using Mendelian Randomisation (STROBE-MR): Explanation and Elaboration. BMJ. 2021;375:n2233.
